# Personalized estimates of brain cortical structural variability in individuals with Autism spectrum disorder: the predictor of brain age and neurobiology relevance

**DOI:** 10.1186/s13229-023-00558-1

**Published:** 2023-07-28

**Authors:** Yingying Xie, Jie Sun, Weiqi Man, Zhang Zhang, Ningnannan Zhang

**Affiliations:** 1 Department of Radiology and Tianjin Key Laboratory of Functional Imaging, Tianjin Medical University General Hospital, No. 154, Anshan Road, Heping District, Tianjin, 300052 China; 2 Department of Radiology, Tianjin First Central Hospital, Tianjin, 300192 China

**Keywords:** Autism spectrum disorder, Person-based similarity index, Gray matter volume, Brain age, Gene expression, Cognition

## Abstract

**Background:**

Autism spectrum disorder (ASD) is a heritable condition related to brain development that affects a person’s perception and socialization with others. Here, we examined variability in the brain morphology in ASD children and adolescent individuals at the level of brain cortical structural profiles and the level of each brain regional measure.

**Methods:**

We selected brain structural MRI data in 600 ASDs and 729 normal controls (NCs) from Autism Brain Imaging Data Exchange (ABIDE). The personalized estimate of similarity between gray matter volume (GMV) profiles of an individual to that of others in the same group was assessed by using the person-based similarity index (PBSI). Regional contributions to PBSI score were utilized for brain age gap estimation (BrainAGE) prediction model establishment, including support vector regression (SVR), relevance vector regression (RVR), and Gaussian process regression (GPR). The association between BrainAGE prediction in ASD and clinical performance was investigated. We further explored the related inter‐regional profiles of gene expression from the Allen Human Brain Atlas with variability differences in the brain morphology between groups.

**Results:**

The PBSI score of GMV was negatively related to age regardless of the sample group, and the PBSI score was significantly lower in ASDs than in NCs. The regional contributions to the PBSI score of 126 brain regions in ASDs showed significant differences compared to NCs. RVR model achieved the best performance for predicting brain age. Higher inter-individual brain morphology variability was related to increased brain age, specific to communication symptoms. A total of 430 genes belonging to various pathways were identified as associated with brain cortical morphometric variation. The pathways, including short-term memory, regulation of system process, and regulation of nervous system process, were dominated mainly by gene sets for manno midbrain neurotypes.

**Limitations:**

There is a sample mismatch between the gene expression data and brain imaging data from ABIDE. A larger sample size can contribute to the model training of BrainAGE and the validation of the results.

**Conclusions:**

ASD has personalized heterogeneity brain morphology. The brain age gap estimation and transcription-neuroimaging associations derived from this trait are replenished in an additional direction to boost the understanding of the ASD brain.

**Supplementary Information:**

The online version contains supplementary material available at 10.1186/s13229-023-00558-1.

## Background

Autism spectrum disorder (ASD) is a neurodevelopmental condition characterized by diminished social interactions, impaired communication, and repetitive and/or restrictive behaviors [1]. Most previous neuroimaging studies have reported several morphometrical brain alterations in ASD, such as subcortical brain abnormalities of striatal structures and amygdala, as well as more specific cortical effects in the frontal and temporal lobes [2–5]. However, the findings represent group comparisons that may not apply to individual patients. The current emphasis on precision psychiatry has shifted the focus of analysis from groups to single individuals [6, 7]. Brain morphometry shows marked inter-individual variation in the general population that reflects the specific genetic and environmental background of each person. The ASD has been considered as a whole group in the analysis. And yet, individuals in the ASD group are characterized by high phenotypic heterogeneity.

The person-based similarity index (PBSI) is used to capture the brain structural profile similarity between each participant and that of other group members by using MRI data, which can quantify variations of brain structures at an individual’s level. Previous studies assessing the brain structural heterogeneity in ASD have focused more on classifying ASD subtypes with distinct neuroanatomical differential patterns based on machine learning methods [8–10]. In this study, PBSI was employed to evaluate the discretization of brain morphometric changes in each individual, and further capture the overall pattern of neuroanatomical heterogeneity, that may produce by the disease mechanism. The PBSI has been previously demonstrated as a biologically and functionally meaningful brain measure, and has been used to quantify brain structural heterogeneity and its association with cognitive dysfunction in neuropsychiatric patients [11–15] with high translational potential and stability [16, 17]. In addition, PBSI is a heritable index and is robust to variation in neuroimaging parameters, sample composition, and regional contribution [17]. Thus, PBSI was used in our study to examine the brain structural heterogeneity in ASD, and its correlations with cognitive measures and neurobiology relevance.

Brain development is a complex process that occurs throughout childhood, adolescence, and early adulthood [18], and identifying typical and atypical brain developmental trajectories is critical for the assessment and intervention of mental disorders such as ASD [19]. MRI indices are used to develop biomarkers and establish the trajectories of brain development [20, 21]; of these, brain age gap estimation (BrainAGE) is promising and has been reported in diseased populations [22–24]. For ASD, the positive BrainAGE values indicate advanced structural brain maturation, whereas negative values indicate delayed structural brain maturation. In addition, BrainAGE was associated with ASD severity in communication and social interaction abilities [23]. Inter-individual variation in brain morphometry is very important for cognitive neuroscience. The PBSI score has been reported to correlate significantly with age, sex, and cognition assessment [11, 13], and it remains an open question whether the PBSI score can be combined with BrainAGE calculation and potentially provide more efficient brain information.

Gene hunting is on the path to precision medicine for ASD [25], and brain morphology has been reported to be highly heritable [26]. However, the neurobiological changes underlying these brain structural differences are not well understood. In order to gain further insights into the similarity between the brain structural profiles, we employ a virtual histological approach. The Allen Human Brain Atlas (AHBA) provided brain gene expression data, and spatial correlations between expression and brain structural profiles can be performed to evaluate the transcription-neuroimaging associations [27, 28]. This approach has been reported to characterize the neurobiology of group differences in cortical thickness and volume in ASD [29, 30].

**Fig. 1:**
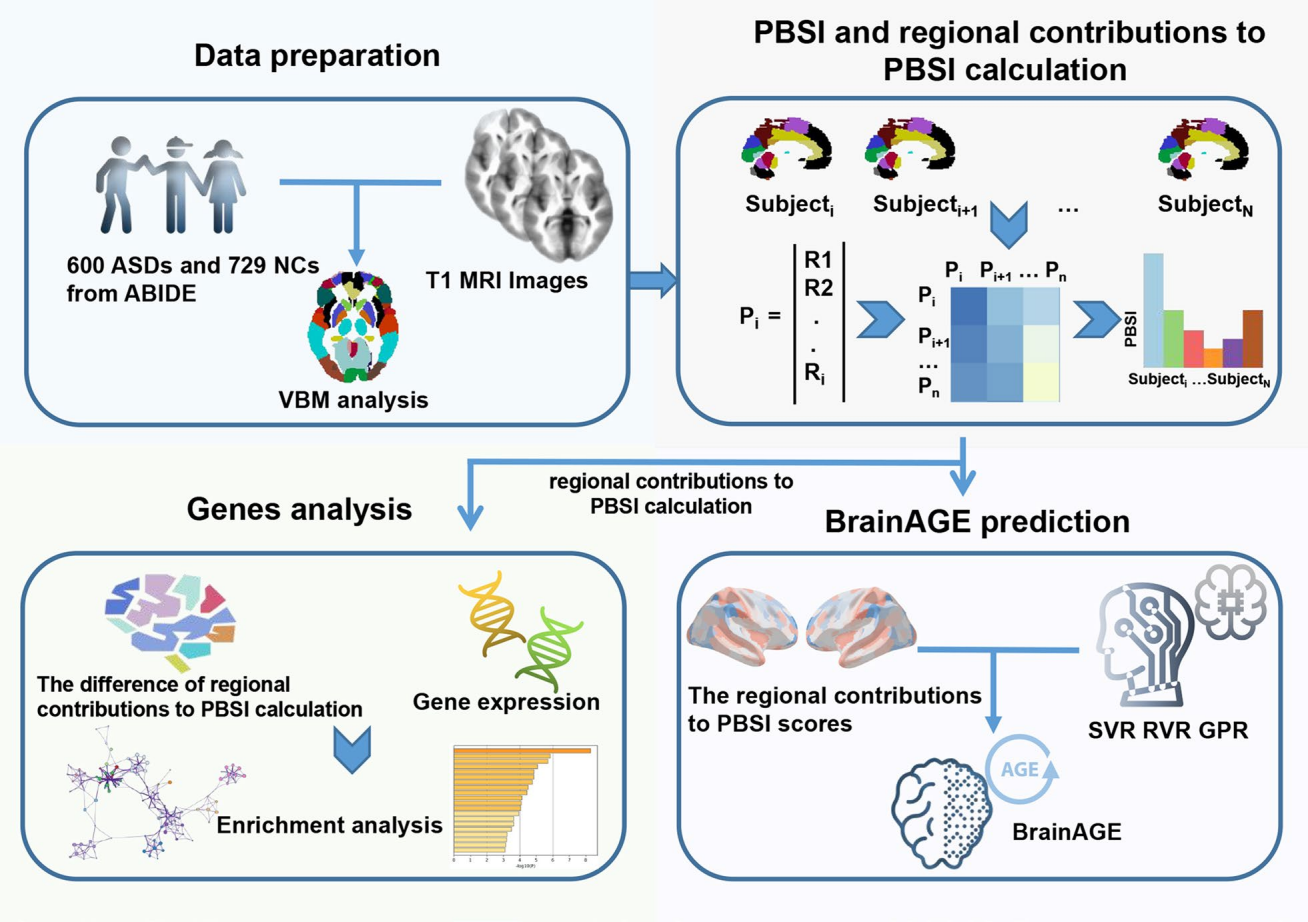
The flowchart of the study design. A total of 600 ASDs and 729 NCs from Autism Brain Imaging Data Exchange (ABIDE) were included in the study, and the gray matter volume (GMV) was calculated based on the voxel-based morphometry (VBM) analysis. Then, the person-based similarity index (PBSI) scores for GMV were calculated in each subject. Based on the regional contributions to PBSI scores, BrainAGE models were constructed using different machine learning methods, and the correlations between the difference of regional contributions to PBSI scores and gene expression were explored. *Abbreviation* ABIDE: Autism Brain Imaging Data Exchange; ASD, Autism spectrum disorder; GPR, Gaussian process regression; MRI, magnetic resonance imaging; NC, normal control; PBSI, person-based similarity index; RVR, relevance vector regression; SVR, support vector regression; VBM, voxel-based morphometry

In this study, the PBSI score was calculated using the gray matter volume (GMV) data of 600 ASDs and 729 normal controls (NCs) from Autism Brain Imaging Data Exchange (ABIDE) data, and regional contributions to PBSI score were obtained. We initiated two independent yet synergistic studies to examine variability in brain morphology in ASD children and adolescents. First, through the PBSI platform, we test whether the variability in brain structural profiles and regional brain measures for ASD is different from that for NCs, and explore whether the regional brain measures can be useful to establish a brain age model for ASD-related behavioral dysfunction. Second, the neurobiology of these neuroimaging phenotypes is further investigated by transcription-neuroimaging associations. A systematic flow of the study design is shown in Fig. 1.

## Methods

### ABIDE-I and ABIDE-II participants

The subjects were selected from the ABIDE, and all analyses were in keeping with the standards of Institutional Review Board guidelines [31, 32]. The inclusion criteria for the subject age in this study were no more than 20 years old.

Across ABIDE I and ABIDE II, the structural MRI data were used for neuroimaging analysis, detailed information about the centers and scan procedure and parameters can be found at http://fcon_1000.projects.nitrc.org/indi/abide/. Quality control measures were included: (1) the MRI images with artifacts were excluded, such as motion artifacts and ghost artifacts; (2) the subjects with poor segmentation were excluded; (3) the subjects without full intelligence quotient (FIQ) evaluation were excluded. Finally, 600 ASDs and 729 NCs were recruited for the analyses (ASDs, age: 12.70 ± 3.65, range from 5 to 20 years old, FIQ: 105.64 ± 17.12; NCs, age: 12.20 ± 3.18, range from 6 to 20 years old, FIQ: 112.95 ± 12.70), detailed demographic information for ASDs and NCs is shown in Tables S1–S2 (Additional file 1). The enrollment of subjects and quality control procedures of structural MRI data were shown in Figure S1 (Additional file 1).

### Structural MRI data preprocessing and GMV measures

The structural MRI data were segmented into white matter, gray matter, and cerebrospinal fluid and thus GMV maps were obtained, according to the standard voxel-based morphometry (VBM) pipeline of Statistical Parametric Mapping 12 (SPM12, https://www.fil.ion.ucl.ac.uk/spm/). The template was custom-designed to meet data analysis for children and adolescents with ASD and age-matched NC. The segmented images were further normalized using the Diffeomorphic Anatomical Registration Through Exponentiated Lie Algebra (DARTEL) technique [33]. At last, the normalized images were resampled into 1.5 × 1.5 × 1.5 mm^3^ and smoothed by a Gaussian kernel of 8 mm full width half maximum (FWHM).

### Computation of the PBSI scores

In order to quantify the within-diagnosis similarity of neuroimaging profiles [11, 12], the PBSI scores for GMV were calculated separately in ASD and NC following the steps below. Firstly, the cortical volume profile of each subject was extracted through a parcellation mask (180 areas for each hemisphere) of the cerebral cortex [34]. Secondly, in consideration of ABIDE being a multi-site data, Combat harmonization was used to remove the center bias [35], with age, sex, and total intracranial volume as covariates. After that, we calculated the inter-individual Spearman correlation coefficient according to the volume profile of each subject. Consequently, the correlation coefficients were averaged to generate the PBSI score for the volume of each brain area for each subject (Figure S2, Additional file 1). High values of PBSI score indicate that the imaging profile of any one group member accurately predicts the profiles of all other members, whereas low values signify low consistency among the imaging profiles of the group members.

### Contribution of regional brain measures to the PBSI

The regional contributions to the PBSI score were calculated through the leave-one-out strategy. The PBSI score of each subject was recalculated by leaving out one brain area measure at a time. The regional contribution for brain area *i* was finally defined as the absolute difference between the original PBSI score and the recalculated PBSI score with the removal of *i* from the calculation. Group difference comparison was performed in each brain area to explore the difference in regional contributions to PBSI score between ASDs and NCs. Age and sex were regarded as covariates and Bonferroni’s method was used to correct (*P* < 0.05/360).

### Construction of the brain age model and validation

Three conventional machine learning methods were used in NCs, including support vector regression (SVR), relevance vector regression (RVR), and Gaussian process regression (GPR). SVR model by mapping the data to a high dimension finds a flat hyperplane that minimizes the deviation of the training data and calculates the regular hyperparameter *C* to reduce overfitting [36]. The grid search method was used for hyperparameter search for *C* over the search space 2^–7^, 2^–5^, 2^–3^, 2^–1^, 1, 2, 2^3^, 2^5^, and 2^7^ in a tenfold nested CV (stratified by age). RVR is a Bayesian sparse learning approach that prunes the basic functions with weak precision, and its parameter optimization is not necessary for contrast to SVR [37]. RVR was implemented for expectation maximization with a precomputed linear kernel. GPR is a nonparametric Bayesian approach by adjusting hyperparameters to maximize the likelihood of the training data [38]. As suggested previously [39], we used a linear kernel to train the machine learning model in this study, the scikit-learn library was used to perform our experiments [40, 41].

The brain age model was constructed based on the regional contributions to PBSI score in NCs. Tenfold cross-validations were utilized to set out the brain age model and estimate age estimation accuracy. To make the age distribution of the training and validation sets similar, and to further evaluate the reproducibility of the model, a strategy of stratification by age was used and the data distribution was shuffled 10 times. After that, we also obtained the predicted age of the ASDs using the pre-trained models based on the NCs. Finally, the mean predicted age of each individual in the validation set was taken as brain age, and then BrainAGE was calculated as the difference between predicted and chronological age. Based on BrainAGE, ASDs were divided into two groups, in consideration of the potential generalization errors, the standard deviation (SD) of brain age was calculated, and the delayed development (DED) group was defined as brain age + 0.5SD < chronological age, and the premature development (PRD) group was defined as brain age-0.5SD > chronological age.

The correlation coefficient, mean absolute error (MAE), and root mean square error (RMSE) were calculated in the validation set of each trained model, and the mean and standard deviation of these indicators for the 10 shuffles were obtained. Besides, the correlations between predicted and chronological age in each kind of model were calculated. The paired Student’s *t* test was performed for the MAE of the three models, corrected by Bonferroni’s method (*P* < 0.05/3). According to the predicted performance of three models (SVR, RVR, and GPR), the model with the highest predicted performance was used in further analyses.

### Gene expression data processing

The normalized microarray gene expression data of 2 donated brains with the whole brain coverage were obtained from the AHBA (http://human.brain-map.org) [27, 28]. The preprocessing of gene expression data followed a newly proposed pipeline based on AHBA data [42]. Briefly, probes were reassigned to genes by utilizing the latest NCBI database, probes were further excluded with expression intensity lower than the background signal in more than 50% of samples. After that, probes were selected with the highest correlation with the RNA-seq data and the scaled robust sigmoid method was used in data normalization. The Montreal Neurological Institute (MNI) coordinate of each sample was provided by the AHBA, and the samples were included in each brain area if the largest Euclidean distance calculated between each sample and voxel in the mask (180 areas for each hemisphere) was within 3 mm [42], the average expression of samples in each mask was regarded as the expression of each brain area. Finally, 10,185 candidate genes were obtained after data preprocessing and the gene expression values in each brain area were obtained.

### Transcription-neuroimaging association across brain regions

The mean regional contributions to PBSI score in each brain area can be obtained in ASDs and NCs, and Δregional contributions to PBSI score were defined as the difference between the mean regional contributions to PBSI score of ASDs and NCs (ASDs-NCs). Spearman correlation across brain areas analysis was performed to evaluate the associations between the Δregional contributions to PBSI score and the gene expression, to explore the genes that potentially regulate the difference in brain variation between ASDs and NCs. The Metascape (https://metascape.org/gp/index.html) was used for gene functional annotations and cell-type enrichment analysis which is based on over 40 bioinformatics knowledgebases (terms across different ontology sources, including KEGG Pathway, GO Biological Processes, Reactome Gene Sets, Canonical Pathways, Cell-Type Signatures, CORUM, TRRUST, DisGeNET, PaGenBase, Transcription Factor Targets, WikiPathways and COVID) [43]. The Benjamini and Hochberg FDR (BH-FDR, *q* < 0.05) method was used to correct multiple testing.

### Statistical analysis

The PBSI scores were calculated separately in each group (ASDs and NCs), and an appropriate test based on data distribution was used to evaluate the difference in PBSI scores between ASDs and NCs (*P* < 0.05). The age and sex were regards as covariates for all group comparisons. A two-sample *t* test was used for normal distribution data, and a Wilcoxon rank sum test was used for non-normally distribution data. The Spearman correlations were performed to evaluate the relationship between PBSI scores and age, and between PBSI scores and cognition scores (*P* < 0.05). For association with cognition scores, age and sex were taken as covariates. The Autism Diagnostic Interview-Revised (ADI-R) is considered one of the “gold standard” assessment measures in the evaluation of ASD [44]. For cognition evaluation, the FIQ, the Restricted, Repetitive, and Stereotyped Patterns of Behavior (ADI-R-RRB) scores, Reciprocal Social Interaction scores (ADI-R-SOC) and Abnormalities in Communication Verbal scores (ADI-R-VER) from ADI-R were included in the analyses [45].

To explore the effects of the DED and PRD group on the cognitive scores (i.e., FIQ, ADI-R-RRB scores, ADI-R-SOC scores, and ADI-R-VER scores) and BrainAGE, an appropriate test based on data distribution was performed between the DED group and the PRD group in cognition evaluations.

## Results

### Clinical features and demographics analysis

**Fig. 2:**
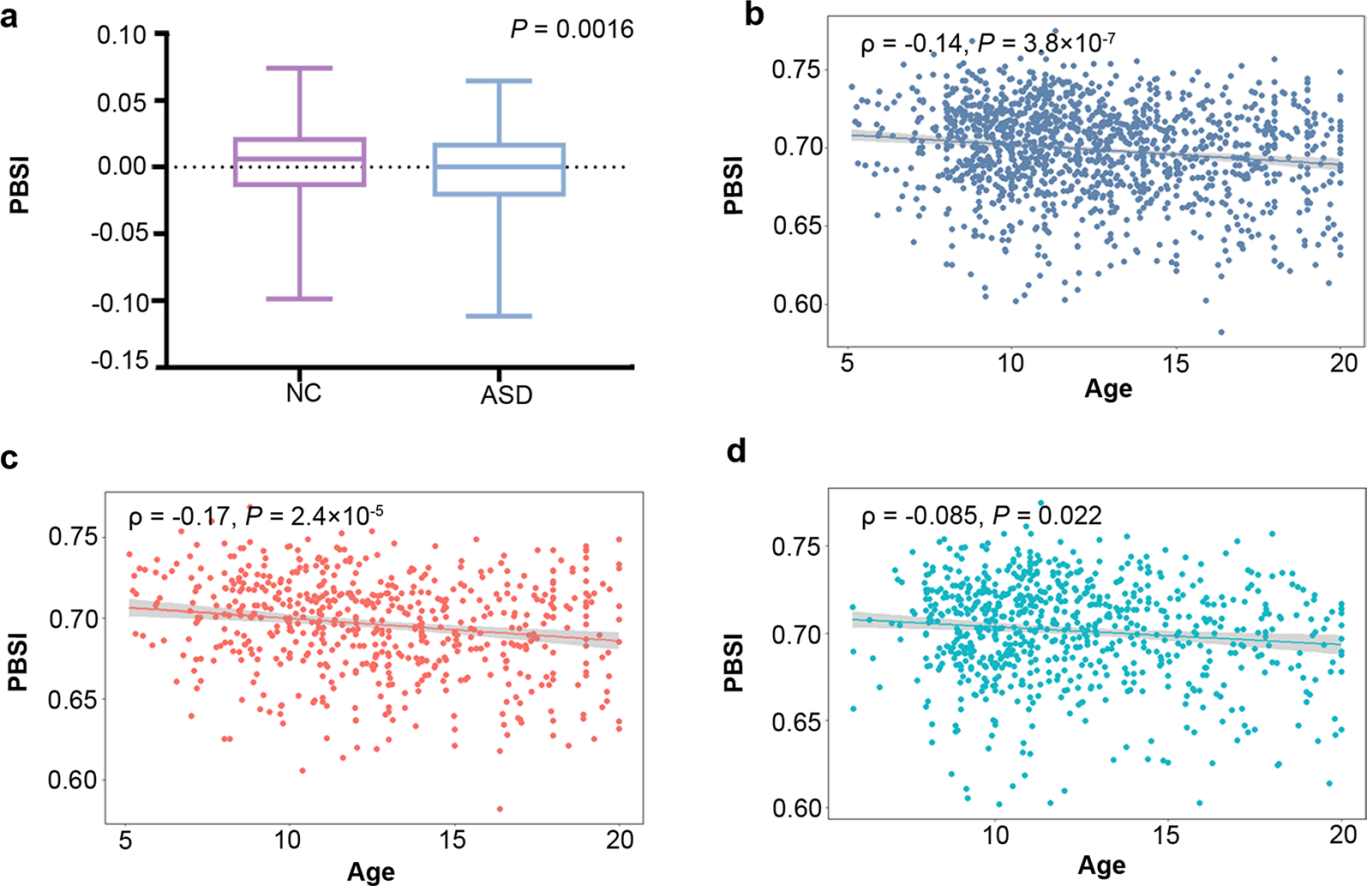
The difference in the PBSI score between subgroups and the associations between PBSI and age. **a** The difference in the PBSI scores between ASDs and NCs (with age and sex as covariates). **b–d** Correlations between the PBSI score and age in **b** all subjects, **c** ASDs, and **d** NCs. *Abbreviation* ASD, Autism spectrum disorder; NC, normal control; PBSI, person-based similarity index

Compared to NCs, patients with ASDs showed lower PBSI scores (ASD, median (Q1, Q3) = 0.70 (0.68, 0.72); NC, median (Q1, Q3) = 0.70 (0.68, 0.72); *P* = 1.6 × 10^−3^, Fig. 2a). Besides, patients with ASDs also showed lower PBSI scores than NCs in ABIDE I and ABIDE II, respectively (Figure S3, Additional file 1). The PBSI scores showed significant negative correlations with age in total subjects (ρ = − 0.14, *P* = 3.8 × 10^–7^, Fig. 2b), in patients of ASDs (*ρ* = − 0.17, *P* = 2.4 × 10^–5^, Fig. 2c), and in NCs (*ρ* = − 0.085, *P* = 2.2 × 10^–2^, Fig. 2d). The significant correlations were also found in subgroup analyses for ABIDE I and ABIDE II (ABIDE I, *ρ* = − 0.18, *P* = 6.72 × 10^–6^; ABIDE II, *ρ* = − 0.13, *P* = 6.47 × 10^–4^, Figure S4, Additional file 1). However, no significant difference was found in the PBSI score between males and females (*P* = 0.92), and no significant relationship was found between the PBSI score and FIQ, ADI-R-SOC, ADI-R-VER, and ADI-R-RRB in neither total subjects nor subgroups with age and sex as covariates (Figure S5, Additional file 1).

**Fig. 3:**
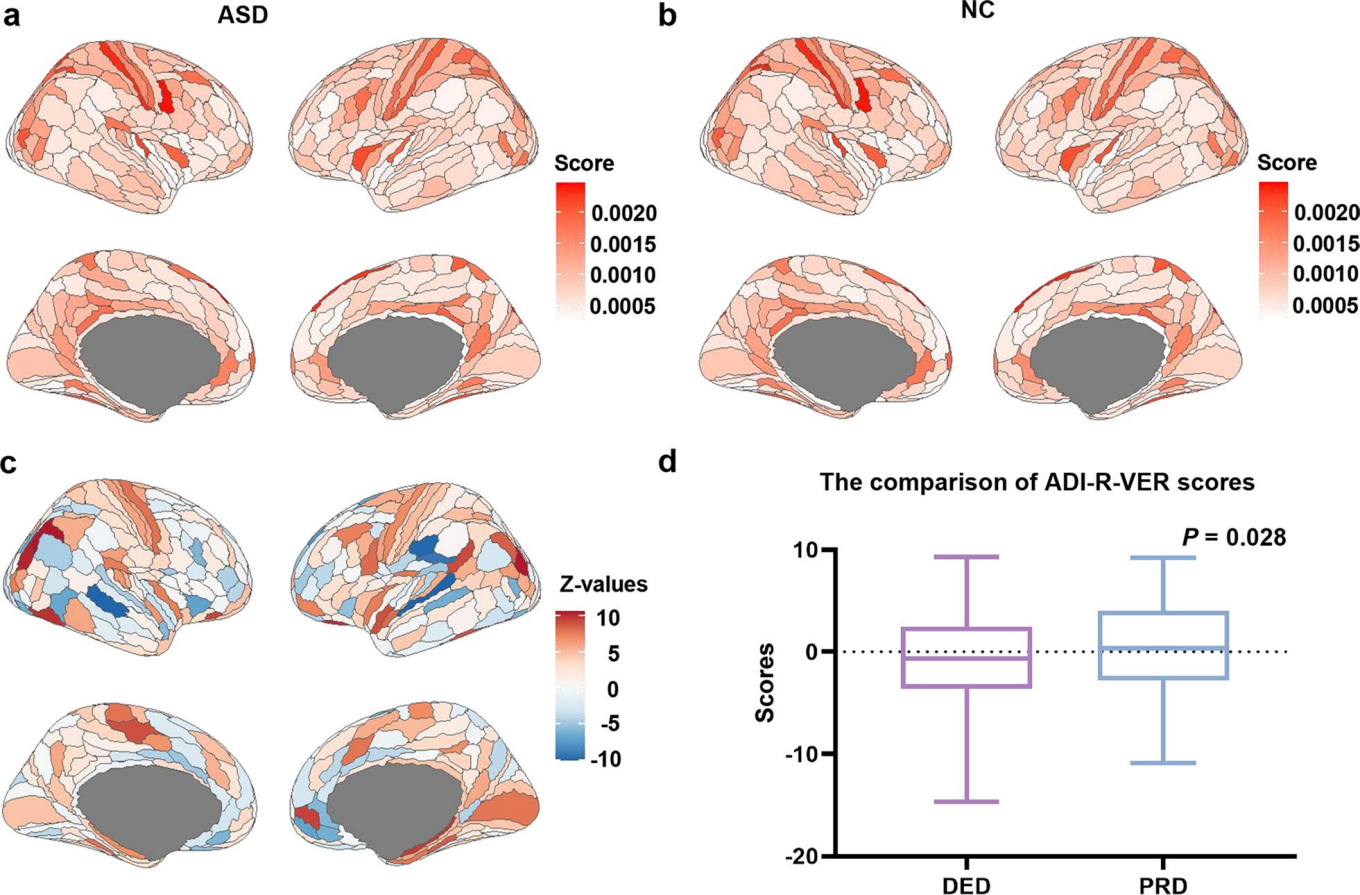
The distribution of average regional contributions to PBSI score in ASDs and NCs and between-group comparisons. **a–b** Distribution maps of average regional contributions to PBSI score in **a** ASDs and **b** NCs. The color bar represents the average value of regional contributions to PBSI score. **c** Distribution maps of group differences of regional contributions to PBSI score. The color represents the Z values (in comparison to NCs). **d** The comparison of ADI-R-VER scores between the PRD group and DED group in ASDs with age and sex as covariates. The y-axis represents the ADI-R-VER scores. *Abbreviation*: ADI-R-VER, the Abnormalities in Communication Verbal scores from the Autism Diagnostic Interview-Revised evaluation from the Autism Diagnostic Interview-Revised evaluation; ASD, Autism spectrum disorder; DED, the delayed development group; NC, normal control; PBSI, person-based similarity index; PRD, the premature development group

On behalf of the contribution of brain morphology variability, the mapping of average regional measures of PBSI score in ASDs is shown in Fig. 3a, and NCs are shown in Fig. 3b. Thus, compared to NCs, 126 brain areas showed significant differences in ASDs (*P* < 0.05/360, Fig. 3c), which were dispersedly distributed in the cerebral cortex. Especially, among 126 brain areas, half of them belong to the somatomotor network (*n* = 24) and default network (*n* = 23) and visual network (*n* = 22). The detailed description of 126 brain areas is in Table S3 (Additional file 2).

### Validation and optimization of three brain age models

RVR model achieved the best performance compared to the other two models (RVR vs GPR, *P* = 0.01; RVR vs SVR, *P* = 0.07). Besides, the RVR model showed the highest correlation with chronological age in ASDs (*ρ* = 0.69, *P* < 0.001) and NCs (*ρ* = 0.58, *P* < 0.001), compared with SVR (ASDs, *ρ* = 0.59, *P* < 0.001; NCs, *ρ* = 0.48, *P* < 0.001) and GPR (ASDs, *ρ* = 0.49, *P* < 0.001; NCs, *ρ* = 0.49, *P* < 0.001) (Figure S6, Additional file 1), so BrainAGE predicted by RVR were used in further analyses. The distribution of BrainAGE (the difference between predicted age and chronological age) predicted by RVR in ASDs and NCs is shown in Figure S7 (Additional file 1).

Besides, in consideration of the potential effects of site on age, we regressed age with the site as a covariate, and used the site-removed age to retrain the brain age model. Spearman correlations were performed between the brain age predicted based on true chronological age and site-removed chronological age. The results showed highly consistent correlations in both ASDs (*ρ* = 0.89, *P* < 0.001) and NCs (*ρ* = 0.87, *P* < 0.001) (Figure S8, Additional file 1). Furthermore, in order to address the potential site effects in covariance, we also added CovBat harmonization [46] as a sensitivity analysis to provide validations. Spearman correlations were performed between brain age predicted by ComBat-Harmonized data and CovBat-Harmonized data and showed high correlations (*ρ* = 0.78, *P* < 0.001), and the Bland–Altman analysis also revealed a good agreement for the predicted brain ages based on the two methods (Figures S9 and S10, Additional file 1).

### BrainAGE prediction and clinical measures

Patients of ASDs were divided into a DED group (i.e., brain age + 0.5SD < chronological age, deviation < 0, *n* = 209) and a PRD group (i.e., brain age − 0.5SD > chronological age, deviation > 0, *n* = 246), according to BrainAGE, which is calculated as the difference between predicted and chronological age. Especially, after regressing age and sex, the PRD group showed significantly higher ADI-R-VER scores than the DED group (*P* = 0.028, two-sample *t* test) (Fig. 3d), which informed the PRD subjects had more severe ASD clinical symptoms than the DED group. However, there was no significant difference between the DED group and PRD group in FIQ (*P* = 0.25), ADI-R-SOC scores (*P* = 0.12), and ADI-R-RRB scores (*P* = 0.13).

### Genes associated with PBSI

**Fig. 4:**
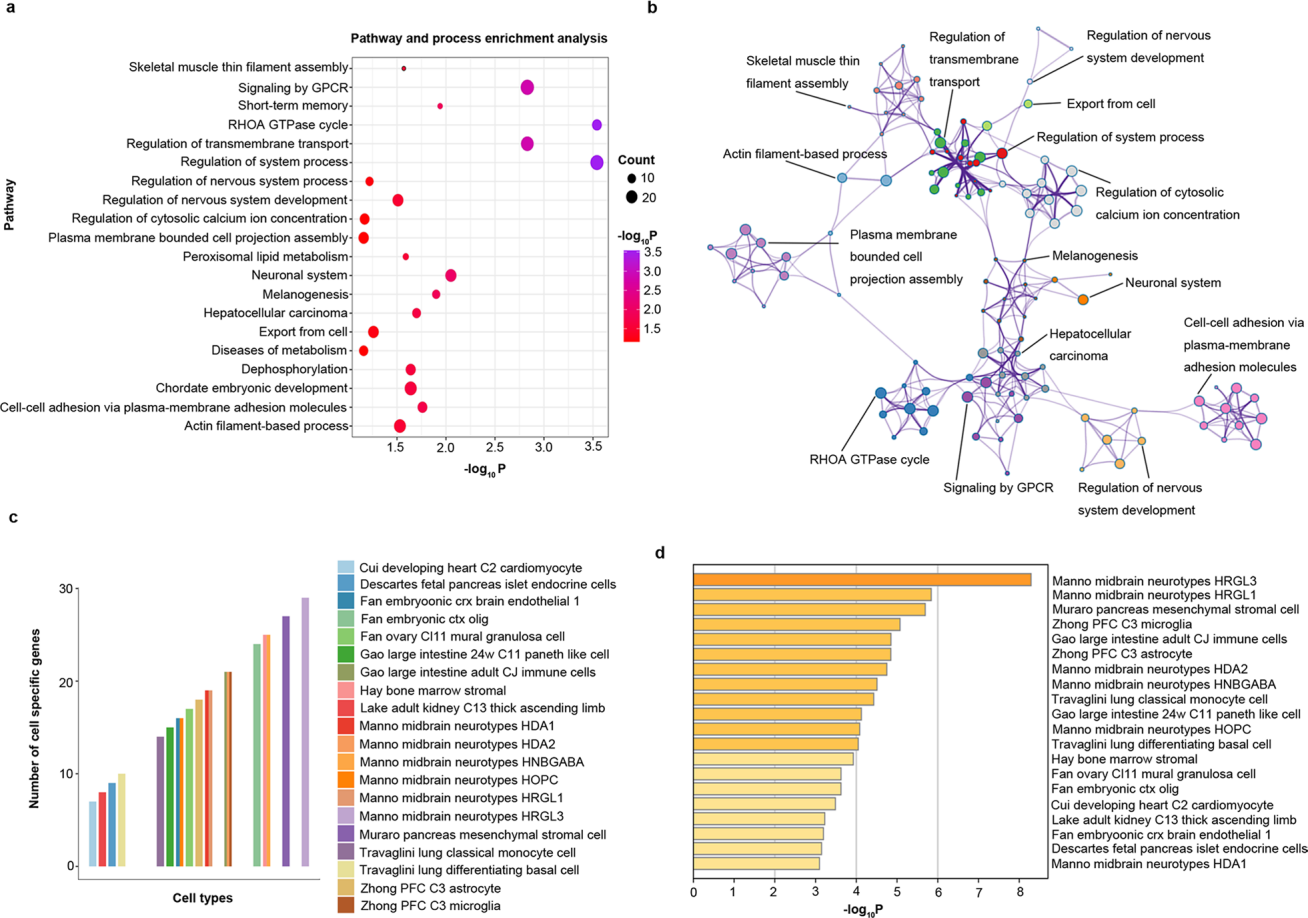
Pathway annotations of 430 genes and the cell-types enrichment analyses. **a** For each item, the globule size reflects the gene numbers, and the globule color represents significance. **b** The clusters of pathways. Each color demonstrates one kind of cluster. **c–d** The cell-type enrichment analyses of 430 genes. **c**. The y-axis represents the number of genes enriched in each cell type, and the colors represent different kinds of cell types. **d** The x-axis represents the -log_10_P value of enrichment analysis in each cell type

Finally, 430 genes showed significant associations with Δregional contributions to PBSI scores (BH-FDR, *q* < 0.05, Table S4, Additional file 2). The functional enrichment analyses revealed that the genes were associated with pathways like short-term memory, export from the cell, regulation of nervous system development, and regulation of nervous system process. Besides, the cell-type enrichment analyses showed the genes were mostly enriched in cells of manno midbrain neurotypes (HRGL3 and HRGL1) (Fig. 4a–d**).**

## Discussion

In this study, we examined variability in the brain morphology in ASD, at the level of brain structural profiles assessed by using the PBSI, which is a personalized estimate of similarity between GMV profiles of an ASD individual to that of other individuals in the same group. The PBSI score of GMV was negatively related to age regardless of the sample group, and the PBSI score was significantly lower in ASDs than in NCs, indicating greater heterogeneity in disease and line with age increase. The majority of brain regions with between-group differences in regional contributions to brain cortical morphological variation mainly appeared in somatomotor, default, and visual networks. At the level of each brain regional measure, the BrainAGE model was constructed using the RVR model which showed the highest predicted performance in the comparison of the three predicted models. The PRD group in ASD demonstrated more severe abnormalities in communication. In addition, 430 genes were identified in relating inter-regional profiles of gene expression from AHBA with inter-regional profiles of brain cortical morphometric variation between ASDs and NCs. Then, we were interested in the pathways to which the genes belonged that were associated with brain cortical morphometric variation. The pathways, including short-term memory, regulation of system process, and regulation of nervous system process, were dominated by gene sets for manno midbrain neurotypes, indicating the biological relevance to brain cortical morphometric variation.

PBSI was proposed as a novel evaluation for variation in brain structural profiles at the individual level, which can provide more information about inter-individual variability in brain structural profiles, and it has been successfully used in the analyses of mental illness [12]. In our study, the PBSI scores showed a significant difference between ASDs and NCs, which informed us that PBSI is a neuroimaging biomarker that may help to distinguish ASDs and NCs. Besides, PBSI showed significant correlations with ages, both in ASDs and NCs. It has been reported that advancing age is correlated with smaller subcortical volumes and thinner cortices in healthy adults [47–49], and the brain morphometric measures can also be changed with the increase with age [50, 51]. The significant associations with age revealed that person-based measures of brain morphometry can reflect certain age information, laying the foundation for BrainAGE analysis and embedding in the brain developmental maps that may facilitate personalized medicine [21].

Regional contributions to PBSI scores can reflect the variation of brain cortical volume at the level of brain areas. Based on regional contributions to PBSI score, 126 brain areas showed a significant difference between ASDs and NCs, which may play an important role in regulating the development of the human brain nervous system. For example, Brodmann’s areas 3b (3b) has been reported to show volume increases in down syndrome patients [52]. The inferior parietal cortex (e.g., PFop, PGp, PGs) is charged with lots of higher cognitive functions and can integrate information from many sensory modalities [53]. The posterior insula (PoI2) plays a critical role in the decision-making neural network and is associated with delaying gratification [54]. Together, the changes in brain cortex variation in those important brain regions may play a key role in regulating the changes in neuroimage phenotypes in ASD.

According to regional contributions to PBSI score, models for predicting brain age were established, and the RVR model was selected according to the high predictive performance with the lowest RMSE and MAE. MRI-derived brain age has been identified as a comprehensive biomarker of brain health, which can reflect both advanced and resilient aging individuals [55]. BrainAGE can show different deviations in different kinds of mental disorders [56]. In our analyses, based on the BrainAGE, the PRD group showed significantly higher ADI-R-VER scores. Language difficulty is a core symptom of ASD as well as one of the earliest predictors of ASD diagnosis, and many children with ASD can have communication challenges across all language sub-systems [57]. The results suggest that ASDs with premature brain development may have more severe abnormalities in communication.

Gene expression analyses revealed that 430 genes showed significant associations with the difference of average regional contributions to PBSI score between ASDs and NCs. The genes were enriched in short-term memory, regulation of system process, and regulation of nervous system process, and showed significant correlations with manno midbrain neurotypes. It has been reported that ASD is linked to impaired synaptic homeostasis and neuronal changes that emerge in the early period of life [58, 59], and midbrain dopamine (mDA) neurons can be replaced to provide long-term improvement in motor functions in patients with Parkinson’s disease [60]. These findings further prove the role of gene expression in regulating brain morphology in ASD.

### Limitations

Several limitations should be mentioned when interpreting our findings. First, the gene expression data were obtained from postmortem brains, and imaging data were derived from ABIDE with ASD patients and normal controls, there is a gap between expression data and brain image data. In addition, although Combat harmonization was used to remove the center bias, the spatial covariance of site-effect was not considered. Thus, we further provide sensitivity analyses based on CovBat harmonization method to prove the robustness of our results. Furthermore, the sufficient sample size is crucial to model training of BrainAGE, and the results should be further verified in large sample data in the future.

## Conclusions

In summary, this study characterized the differences in the cortical morphological similarity between ASDs and NCs by PBSI and demonstrated a high correlation with age. Furthermore, based on regional contributions to PBSI score, BrainAGE models were constructed, and the PRD group showed more severe communication symptoms. Lastly, 430 genes were found associated with the difference in regional contributions to PBSI scores between ASDs and NCs. The functional enrichment analyses of genes can link underlying molecular perturbations with structural morphology changes in brain cortical for ASD. Our study of brain morphology from epigenetic heterogeneity to molecule perturbations may lead to the development of new types of therapy and personalized approaches to treatment.

## Supplementary Information

**Additional file 1**: Supplementary figures and supplementary tables (Table S1 and Table S2).

**Additional file 2**: Supplementary tables (Table S3 and Table S4).

## Data Availability

The datasets supporting the conclusions of this article are available in the Autism Brain Imaging Data Exchange (ABIDE, http://fcon_1000.projects.nitrc.org/indi/abide/).

## Abbreviations

ABIDE: Autism Brain Imaging Data Exchange
ADI-R-RRB: The Restricted, Repetitive, and Stereotyped Patterns of Behavior scores from the Autism Diagnostic Interview-Revised evaluation
ADI-R-SOC: The Reciprocal Social Interaction scores from the Autism Diagnostic Interview-Revised evaluation
ADI-R-VER: Abnormalities in Communication Verbal scores from the Autism Diagnostic Interview-Revised evaluation
AHBA: Allen Human Brain Atlas
ASD: Autism spectrum disorder
DED: The Delayed Development Group
GMV: Gray matter volume
GPR: Gaussian process regression
NC: Normal control
PBSI: Person-based Similarity Index
PRD: The Premature Development Group
RVR: Relevance vector regression
SVR: Support vector regression
VBM: Voxel-based morphometry
