# Supplementary material for: Personalized estimates of brain cortical structural variability in individuals with Autism spectrum disorder: the predictor of brain age and neurobiology relevance
Source: Mol Autism. 2023 Jul 28;14:27. doi: 10.1186/s13229-023-00558-1 (PMC10375633; doi:10.1186/s13229-023-00558-1)
Supplement: Supplementary file 1 — Additional file 1: Supplementary figures and supplementary tables (Table S1 and Table S2). [file 13229_2023_558_MOESM1_ESM.pdf]

Supplementary Materials

Personalized estimates of brain cortical structural variability in individuals with Autism Spectrum Disorder: the predictor of brain age and neurobiology relevance

Supplementary figures

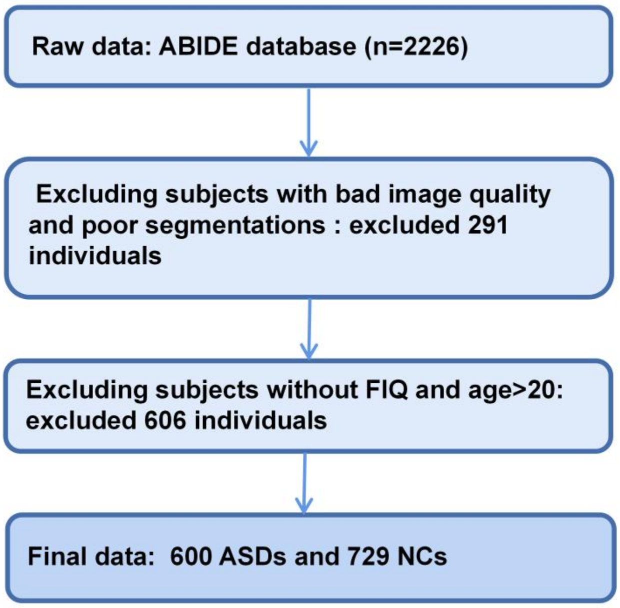

Figure S1. The quality control procedure of neuroimaging data. Abbreviation: ABIDE: Autism Brain Imaging Data Exchange; ASD, Autism spectrum disorder; NC, normal control; FIQ, full intelligence quotient.

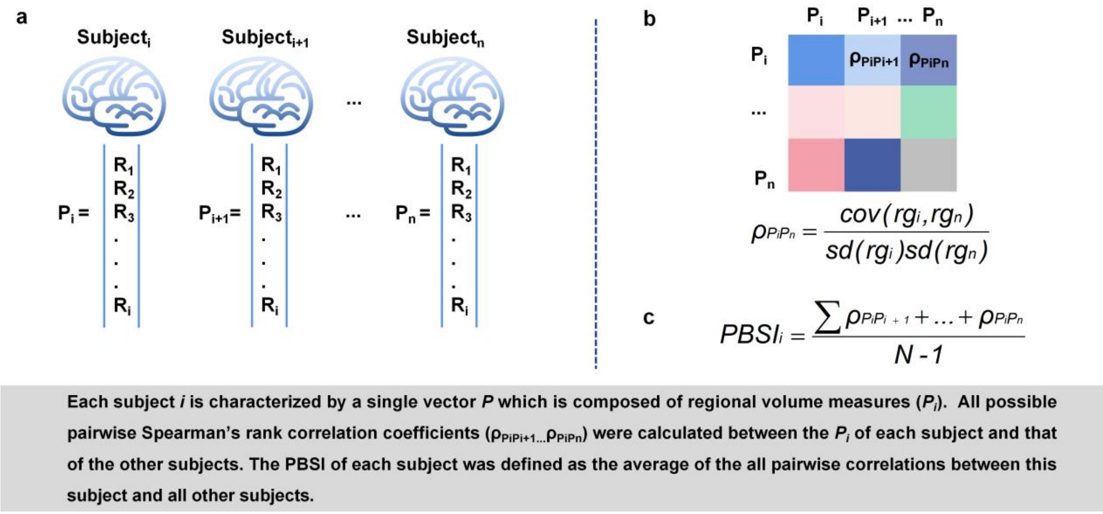

Figure S2. Pipeline for computing the person-based similarity index. Abbreviation: PBSI, person-based similarity index.

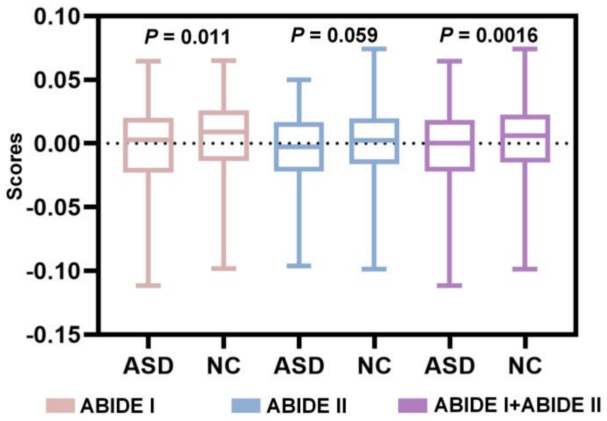

Figure S3. The difference in PBSI score between ASDs and NCs in all subjects, ABIDE I subgroup and ABIDE II subgroup. The y-axis shows the PBSI score. Abbreviation: ASD, Autism spectrum disorder; NC, normal control; PBSI, person-based similarity index.

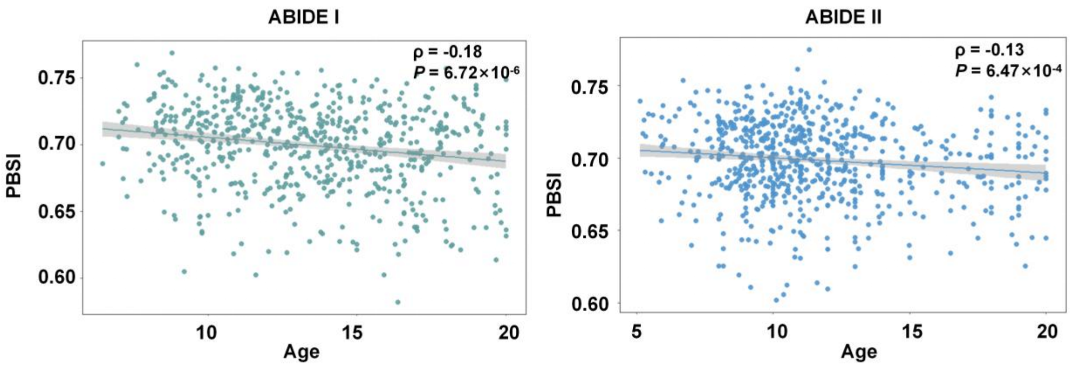

Figure S4. Correlations between PBSI and age in ABIDE I and ABIDE II subgroups. The x-axis shows the age, the y-axis shows the PBSI score. Abbreviation: PBSI, person-based similarity index.

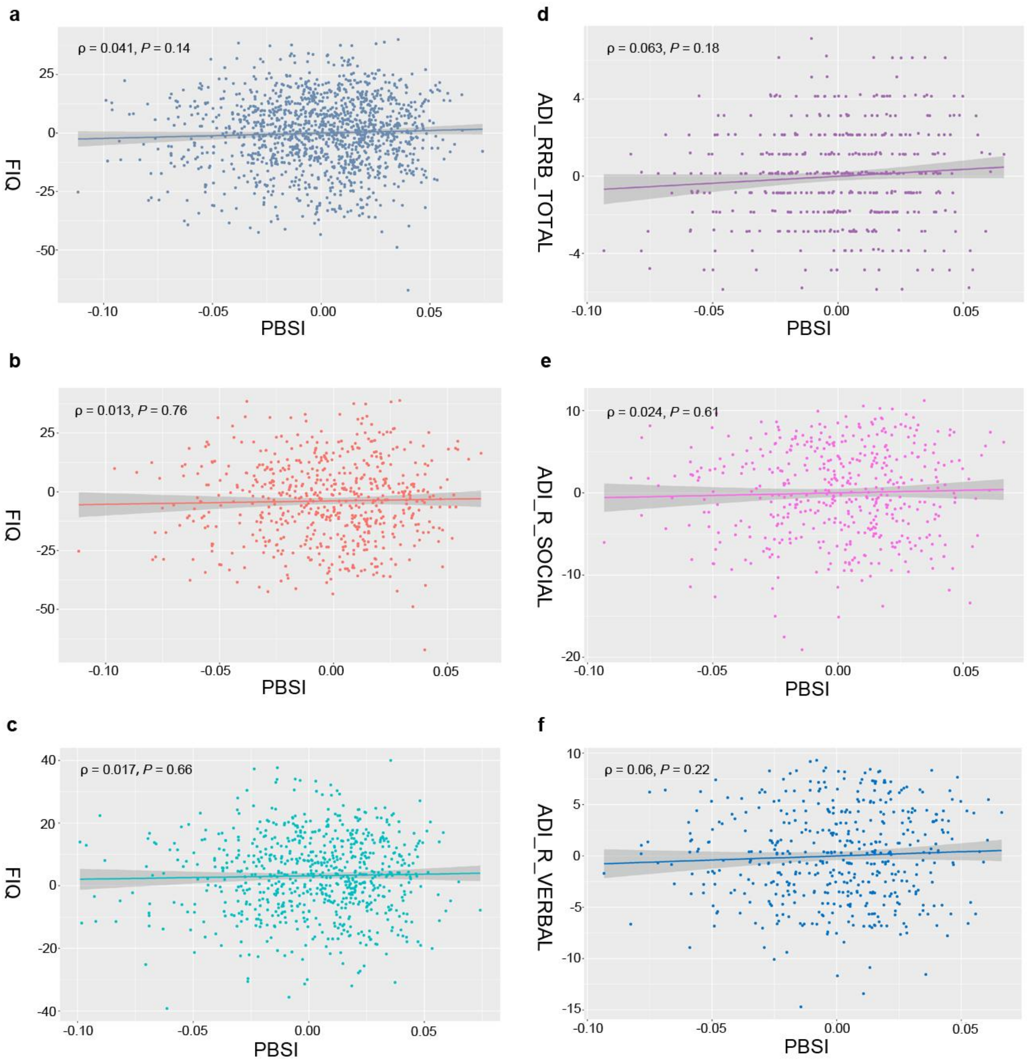

Figure S5. The association between PBSI and FIQ and ADI-R scores. a-c. The association between PBSI and FIQ. a-c. The x-axis shows the PBSI score, and the y-axis shows the FIQ scores in **a)** all subjects **b)** ASDs and **c)** NCs, the x-axis represents the PBSI score, the y-axis represents the FIQ. **d-f.** The association between PBSI and ADI-R scores. The x-axis shows the PBSI score, and the y-axis shows the ADI-R scores (ADI-R-TOTAL, ADI-R-SOCIAL, ADI-R-VERBAL). Abbreviation: ADI-R-RRB, the Restricted, Repetitive, and Stereotyped Patterns of Behavior scores; ADI-R-SOC, Reciprocal Social Interaction scores; ADI-R-VER Abnormalities in Communication Verbal scores; ASD, Autism spectrum disorder; FIQ, full intelligence quotient; NC, normal control; PBSI, person-based similarity index.

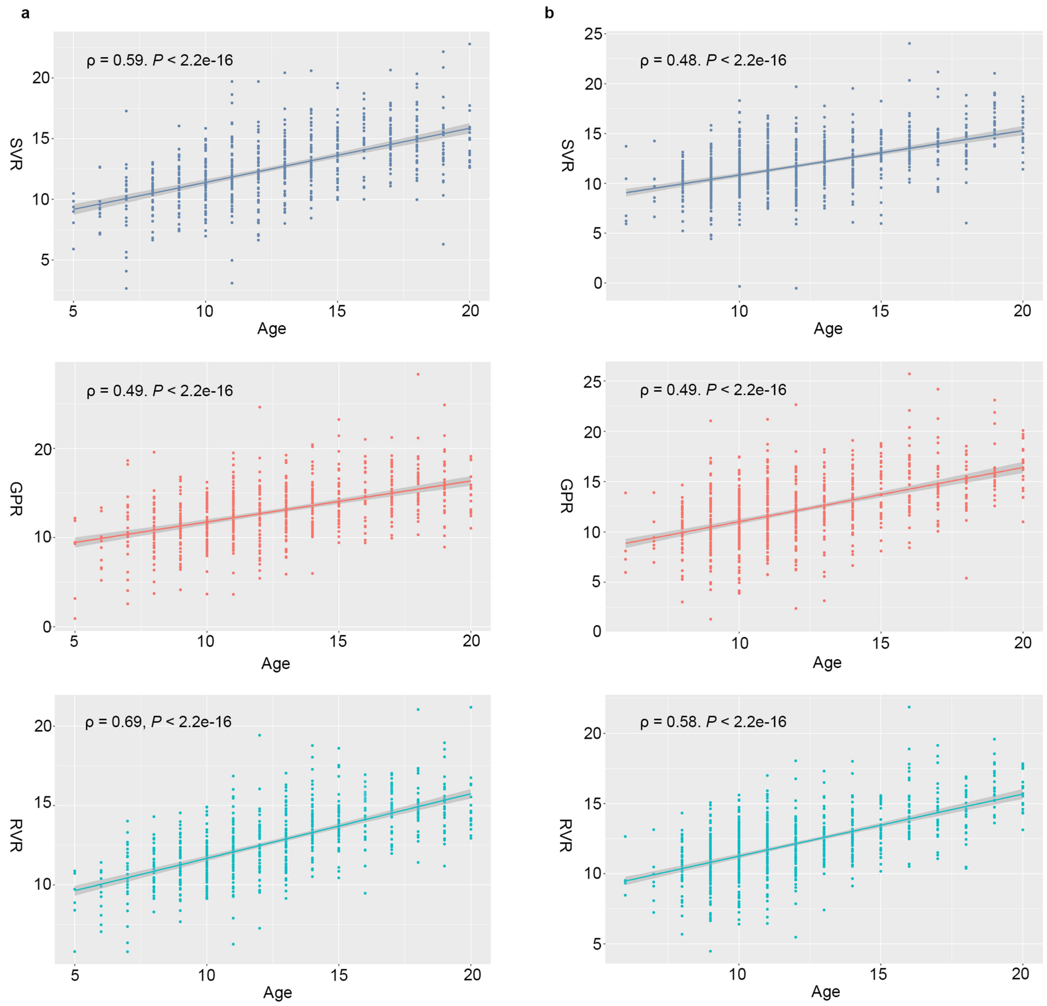

Figure S6. The association between chronological age and brain age predicted by three models (SVR, RVR and GPR). a-b. The association between chronological age (round numbers) and brain age in **a) ASDs** and **b) NCs**. The x-axis shows the chronological age (round numbers), and the y-axis shows the brain age predicted by models. Abbreviation: GPR, Gaussian process regression; RVR, relevance vector regression; SVR, support vector regression.

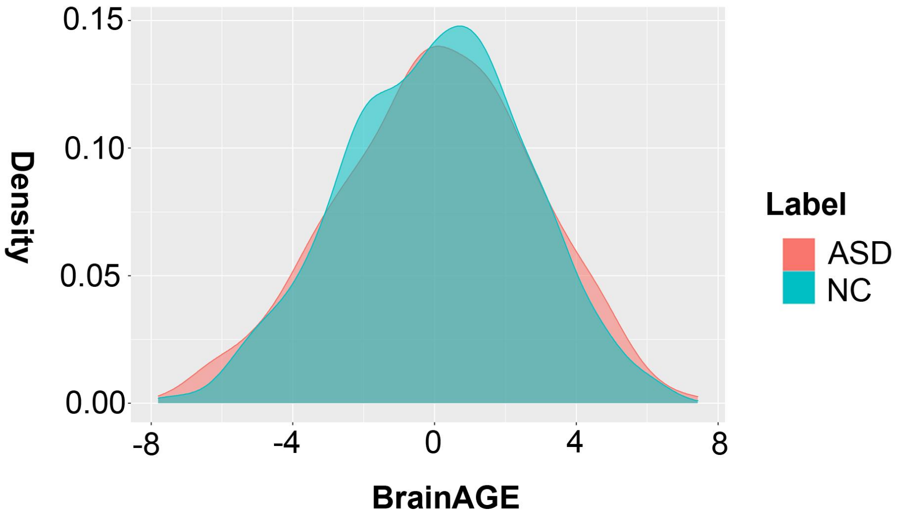

Figure S7. The density map of BrainAGE in ASDs and in NCs. The x-axis represents the BrianAGE, the y-axis represents the density. Abbreviation: ASD, Autism spectrum disorder; NC, normal control.

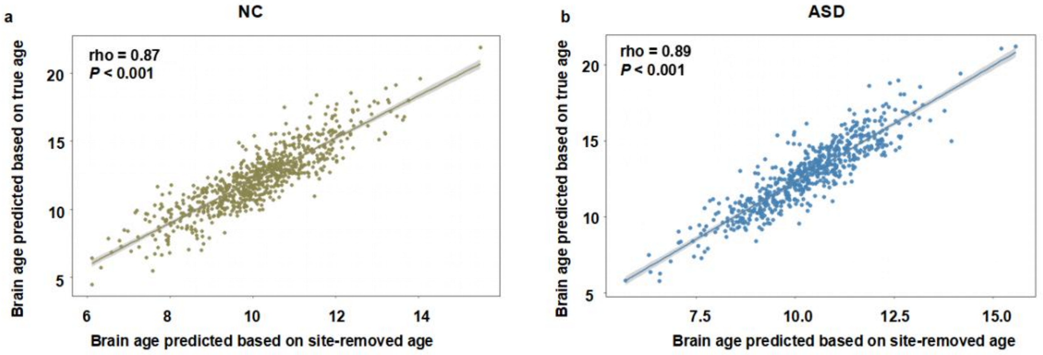

Figure S8. The correlations between brain age predicted based on true chronological age and site-removed chronological age in both ASDs and NCs. Abbreviation: ASD, Autism spectrum disorder; NC, normal control.

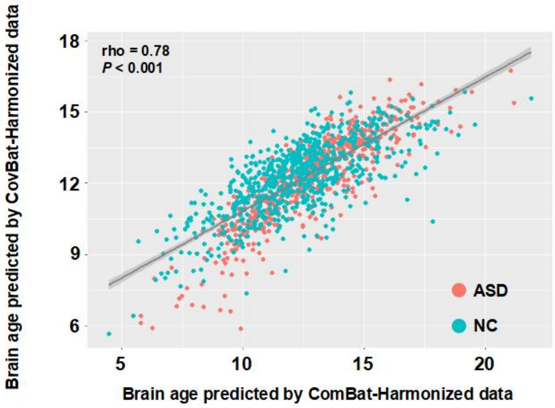

Figure S9. The correlations between brain age predicted based on ComBat-Harmonized data and CovBat-Harmonized data. Abbreviation: ASD, Autism spectrum disorder; NC, normal control.

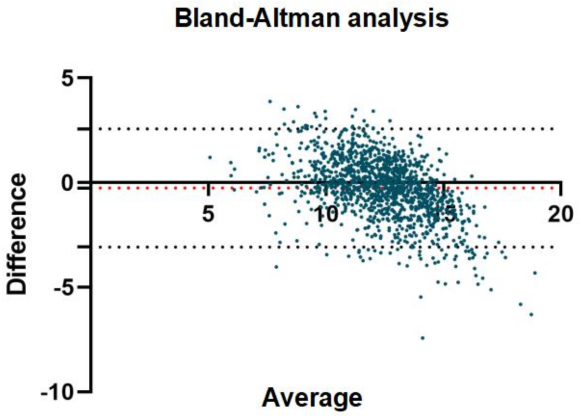

Figure S10. Bland-Altman plot for depicting the agreement between ComBat-Harmonization and CovBat-Harmonization.

Supplementary tables

Table S1. The demographic information of ASD patients.

	ASD					
	**Age (mean)**	**Age (SD)**	**Male**	**Female**	**FIQ (mean)**	**FIQ (SD)**
All	12.702	3.646	514	86	105.640	17.119
ABIDE I	13.617	3.319	254	37	104.829	17.373
ABIDE II	11.840	3.734	260	49	106.405	16.868

Abbreviation: ASD, Autism spectrum disorder; FIQ, full intelligence quotient.

Table S2. The demographic information of normal controls.

	NC					
	**Age (mean)**	**Age (SD)**	**Male**	**Female**	**FIQ (mean)**	**FIQ (SD)**
All	12.197	3.175	541	188	112.946	12.697
ABIDE I	13.245	3.223	274	69	110.424	12.404
ABIDE II	11.267	2.826	267	119	115.187	12.548

Abbreviation: FIQ, full intelligence quotient; NC, normal control.

Table S3. The brain areas showed significant difference in regional contributions to the PBSI score between ASDs and NCs. This table is rendered as an independent Excel file.

Table S4. The 430 genes associated with the difference of PBSI scores between ASDs and NCs. This table is rendered as an independent Excel file.
